# Ipsilateral Arteriovenous Loop and Latissimus Dorsi Free Flap for Knee Reconstruction in an Elderly Patient: A Case Report 

**DOI:** 10.29252/wjps.7.3.377

**Published:** 2018-09

**Authors:** Luis Parra, Juan Andrés, Manuel Robustillo, Carlos García, Israel Iglesias, Antonio Díaz

**Affiliations:** Department of Plastic and Reconstructive Surgery, 12 de Octubre University Hospital, Madrid, Spain

**Keywords:** Ipsilateral arteriovenous, Loop, Latissimus, Flap, Reconstruction

## Abstract

Herein, we report an unusual indication of an arteriovenous (AV) loop with a latissimus dorsi free flap after wound-edge necrosis in an 81 year old patient. The patient underwent multiple revision procedures after total knee arthroplasty and total hip arthroplasty. After a dramatic reduction of femoral bone, a total femoral replacement was performed. The lateral knee incision wound was broke down and the hardware became exposed. Local flaps were not available and a free flap with an ipsilateral AV loop from the great saphenous vein was used to cover the large defect. The functional status of the hip and knee joints was good after 6 months, and enough the patient was able to ambulate without any assistance. The patient did not show any signs of infection.

## INTRODUCTION

Wound-healing problems following total knee arthroplasty (TKA) are challenging for reconstructive surgeons, a delay in diagnosis and management may lead to serious complications even compromising the survival of the limb.^1^ Wound complications and soft tissue loss after total knee replacement can be present in up to 20% of the cases,^2^^,^^3^ adequate management and early soft tissue coverage is critical fort he long term success. In complex cases, total femoral replacement may be the only alternative to disarticulation, being its major drawback the possibility of infection.^1^^,^^4^

The gastrocnemius flap has been traditionally considered the workhorse flap for reconstruction of the upper third of the leg and defects of the knee, but occasionally, more complex techniques, like free flaps may be required to cover large defects. There are still lots of controversies about the optimal management of exposed total joints. It is generally agreed that chronically infected TKAs should be removed, however, aggressive treatment and early local muscle flap coverage has been demonstrated to have good short term results in acute exposures. A close cooperation between orthopaedic and plastic surgeons is crucial, the early identification of threatened total knee arthroplasty exposure and a prompt treatment may avoid potentially devastating consequences for these complex patients.^4^ Herein, we report an unusual indication of an arteriovenous (AV) loop with a latissimus dorsi free flap after wound-edge necrosis in a 81 year old patient.

## CASE REPORT

A 81-year old woman was referred to our department to evaluate a wound dehiscence on her left knee with hardware exposure. The patient did not have any relevant comorbidities and her general status was good. The patient underwent a total left hip arthroplasty at the age of seventy four due to severe osteoarthritis pain that hindered baseline activities. Eight years later, the patient presented to the orthopedics department with a progressive pain in the affected hip, particularly when walking, causing difficulties in deambulation.

An X ray examination revealed an extensive femoral bone loss with displacement of the femoral component, and an MRI showed a femoral pseudo-tumor (bone proliferation). With this finding, a revision total hip arthroplasty was performed with the insertion of a reconstruction ring with cemented dual mobility cups. Three weeks after this last surgery, the patient started with early symptoms of infection (high fever, suppuration, no wound healing and laboratory abnormalities); an attempt at conservative management with intravenous antibiotic, irrigation and suction drainages was unsuccessful and a replacement of the endoprosthesis femur in two stages was planned.

**Fig. 1:**
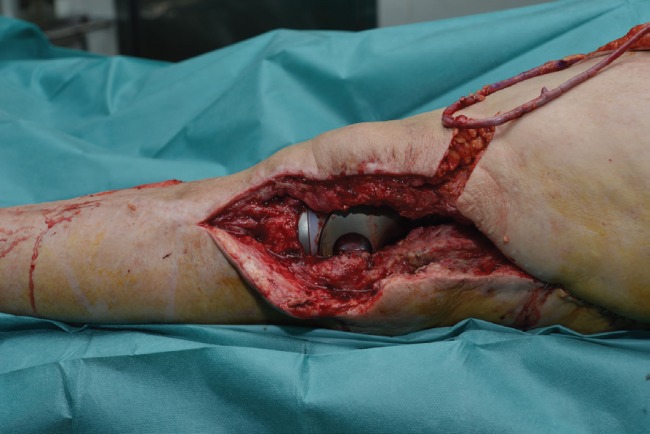
Large lateral defect with hardware exposure

In the first stage, the previous prosthesis was removed with enlarged osteotomy of the anterior tuberosity and a cement spacer with antibiotic was placed. Two months later, the spacer was removed and a new coated silver total femoral prosthesis was placed. The patient was referred to our unit four weeks after the last surgery for the assessment of wound dehiscence to the lateral knee with hardware exposure. The patient was taken to the operating room for retention debridement and wound coverage with a flap. Our first decision was to cover the defect with a lateral gastrocnemius flap, however, the surrounding area was highly scarred, and the gastrocnemius muscle was found to be very atrophic and no suitable to fit the large defect. Figure 1 shows no other local flaps to be large enough to cover the whole defect, so we opted for a free flap.

**Fig. 2:**
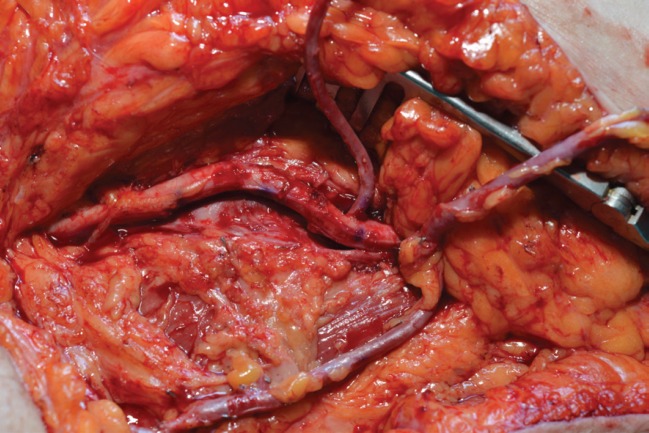
Ipsilateral AV loop with greater saphenous vein, end to side anastomosis to femoral artery

A lack of receptor vessels was evidenced, descending genicular artery was dissected, but small caliber and calcified plaques in the lumen discarded this option. With the need of a suitable and large vessel close to the defect, we opted for an AV loop that was constructed with ipsilateral greater saphenous vein. The femoral artery was found to be very atherosclerotic, but a healthy segment free of calcific plaque on the middle third of the vessel was used to perform the anastomosis of the vein graft in end-to-side fashion (Figure 2).

**Fig. 3:**
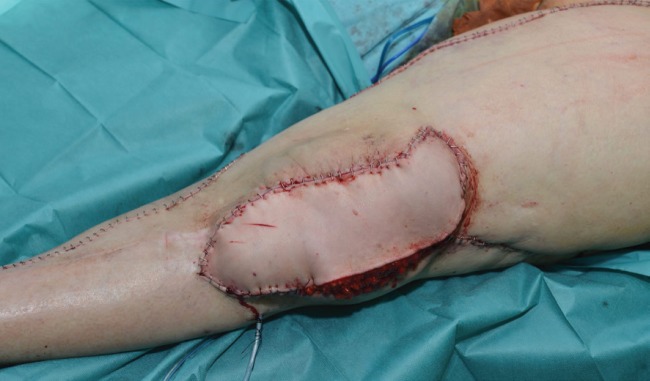
Latissimus dorsi flap after inset

A latissimus dorsi musculocutaneous flap with a large skin paddle was transferred to the lateral knee defect, and the thoracodorsal artery and vein were anastomosed in an end to end fashion to the limbs of the loop. The flap was properly inset to provide adequate bulk and to avoid dead spaces (Figure 3). Donor site was closed primarily. Intraoperatively, no incidences occurred and the flap appeared well perfused. The patient was extubated and transferred to the recovery room in stable condition with standard clinical monitoring. Tissue oximetry system (INVOS CO.) was used 72 hours after the surgery. Three drainages were left, two of them in lower limb placed in lateral knee and lateral hip and one in donor area of the flap. Drainages were retained until output was less than 30 ml per day.

Wound healing properly evolved as well as the patient started rehabilitation during admission. The patient received Daptomycin (700 mg/d iv) and fosfomycin (2 g/6h iv) during 6 weeks, according to the infectious medicine protocol. The patient started walking uneventfully with a walking frame one month after surgery. The patient was enrolled in an outpatient rehabilitation program with close monitoring. At present time, the patient is able to ambulance without the need of assistant devices and there are no signsor symptoms of infection recurrence.

## DISCUSSION

Total joint arthroplasty is a wide-spread orthopaedic procedure, with the majority of patients recovering uneventfully improving joint functionality. The number of primary TKAs and Total hip arthroplasty (THA), as well as revision total arthroplasty, is expected to increase on the upcoming years, especially as patients continue to live longer. Occasionally, total femur replacement is required after previous attempted failed arthroplasties.^6^ High complication rates have been reported with this procedure; specially old patients are at higher risk for developing periprosthetic infection, leading sometimes to unsalvageable total femoral replacement (TFR).^4^^,^^5^^,^^7^

Wound problems after total knee replacement or revision total knee replacement have been reported to be present in up to 20% of the patients,^8^ and can be treated with either prosthesis retention and chronic antibiotic suppression, or with a revision of the prosthesis in one or two stages. The quality of life has been demonstrated to be higher with a functional knee prosthesis than with either arthrodesis or amputation, thus all the efforts should be made to preserve a functioning prosthesis.^4^

Several effective treatment options are available today to manage severe wound complications after total knee arthroplasty but much controversy exists about which is the optimal one,^9^ patient evaluation, careful assessment of associated risks, morbidity and long-term prognosis should be taken into consideration to select the method of closure and coverage. Removal of exposed hardware and inserting a temporary antibiotic impregnated spacer seems to be the method of choice under the presence of a frank chronic infection, however retaining the prosthesis in cases of acute complex defects without clear signs of infection has been proven to be a reliable treatment.^10^

In our patient, a prompt management of a wound dehiscence achieved to retain the total femoral replacement without signs of infection in the midterm evolution. Avoiding the initial formation of biofilms seems to be of great importance for the long term success, the use of drugs that can effectively penetrate and disrupt the biofilm, like rifampicin have demonstrated to be useful to minimize the number of prostheses removed, and to reduce the number of cement spacers or antibiotic beads implanted.^11^

Critically important is the prevention of wound problems in total joint arthroplasty. Particular attention should be paid to vascular anatomy of the knee and care should be taken during dissection of this area. Medial surgical incision should be avoided since major blood supply for anterior knee skin originates medially; full-thickness skin flaps should also be performed during dissection, to avoid compromising the blood supply of the skin. Previous incisions should also be taken into account, preferably using same incisions or the most lateral ones. Early aggressive knee flexion rehabilitation protocols are also discouraged.^12^^,^^13^

Although flap coverage has demonstrated to be a successful procedure to cover exposed knee arthroplasties, with outstanding results in short term evaluation, recent studies show less optimistic results in long term evaluation. Kwiecien *et al.*, reported that, complete eradication of infection associated with hardware exposure, is a complex task, and the long term knee function could be preserved in only 54 percent of cases. They advocate for a proactive and aggressive strategy between the orthopedic surgeon and the plastic surgeon to improve the outcomes.^14^

Patients with threatened total knee arthroplasty exposure should be identified early and treated promptly. Depending on the size of the defect, several options are currently available. When dealing with large defects with hardware exposure, muscular flaps such as latissimus dorsi free flap are probably the best option. They have the potential to improve the trophicity of the neighboring tissue, and the ability to deliver high concentrations of antibiotic and humoral defense factors, reducing the number of infection-related complications in these patients.^4^

Gastrocnemius is still considered the regional workhorse flap for knee reconstruction, but sometimes, as the patient in this reportthis muscle is found to be very atrophic in elderly people or bedridden patients.^12^ The size and the quality of the recipient vessels close to the defect is of utmost importance for the success of microsurgical reconstruction.^15^ In this case, the age of the patient and the scarred tissues around the knee area prevented the used of local receptor vessels. AV loops have demonstrated to be a good solution for these situations.^16^ Large-pedicled free flaps, like the latissimus dorsi used in this case are advocated to avoid vessel caliber mismatch.^17^

This case report highlights the importance of correct diagnosis and early soft tissue reconstruction in patients with risk of wound dehiscence and hardware exposure. It also demonstrates the effectiveness of atypical vascular lesions (AVL) in areas lacking of loco-regional recipient vessels. Age seems not to be a contraindication, but any remarkable comorbidities should be ruled out before attempting this complex procedure. Furthermore, we would like to emphasize the importance of an interdisciplinary work between orthopaedic surgeons and plastic surgeons for the management of the complex wounds. 

## CONFLICT OF INTEREST

The authors declare no conflict of interest.
